# Delays in childhood cancer care at a tertiary care centre in Pakistan: a single-centre study

**DOI:** 10.3332/ecancer.2026.2141

**Published:** 2026-06-09

**Authors:** Ruqayya Manzoor, Hijab Shaheen, Ana Farooq, Nazia Rafique, Nuzhat Yasmeen, Junaid Jamshed

**Affiliations:** 1 Department of Paediatric Oncology, Children’s Hospital, Pakistan Institute of Medical Sciences (PIMS), Islamabad, Pakistan; a https://orcid.org/0009-0003-4650-2751

**Keywords:** childhood cancer, treatment delay, diagnostic delay, health-system delay, paediatric oncology, Pakistan, socioeconomic determinants

## Abstract

**Background:**

Timely diagnosis and initiation of therapy are critical determinants of survival in childhood cancer. In Pakistan, paediatric oncology services are highly centralised, and most children, particularly from rural and low-income households, experience prolonged delays with limited evidence on their cumulative magnitude and determinants. This study determined patient, diagnostic, treatment and health-system delays and identified predictors of prolonged total delay, defined as a cumulative interval exceeding 30 days from symptom onset to treatment initiation, among children with newly diagnosed malignancies.

**Methods:**

Seventy-one children were analysed (median age 5 years; 67.6% male; 71.8% rural). Median total delay was 36 days interquartile range (IQR 49), with 57.7% experiencing prolonged delay. Survival and disease stage were significantly associated with delay duration; children with a total delay of 0–30 days achieved a 12-month event-free survival of 83.3%, which dropped to 60.0% for those with delays >90 days. Furthermore, 50.0% of children in the critical delay group presented with advanced Stages III or IV disease. Prolonged delay was independently associated with rural residence (adjusted OR 2.42), low household income (aOR 3.09), first healthcare contact outside a tertiary centre (aOR 3.96), lack of parental education, absence of caregiver awareness (aOR 2.85) and diagnosis of a solid tumour (aOR 2.67).

**Results:**

Seventy-one children were analysed (median age 5 years; 67.6% male; 71.8% rural). Median total delay was 36 days (IQR 49), with 57.7% experiencing prolonged delay. Patient delay exceeded 14 days in 46.5%, while health-system delay affected 61.9%. Diagnostic delays beyond 21 days occurred in 25.4% and treatment delays were comparatively limited (18.3%). Prolonged delay was independently associated with rural residence (adjusted OR 2.42, 95% CI 1.16–5.03), low household income (< 50,000 PKR; aOR 3.09, 95% CI 1.42–6.72), first healthcare contact outside a tertiary centre (aOR 3.96, 95% CI 1.82–8.63), lack of parental education, absence of caregiver awareness of childhood cancer (aOR 2.85, 95% CI 1.29–6.31) and diagnosis of a solid tumour (aOR 2.67, 95% CI 1.29–5.53).

**Conclusion:**

Delays in childhood cancer care in Pakistan arise from socioeconomic vulnerability, geographic inequity and fragmented health-system pathways at this single-centre site. These delays directly correlate with advanced-stage presentation and reduced survival. Decentralised diagnostics, standardised referral systems, primary healthcare training, social support and community awareness are urgently needed to reduce delays and enhance survival in resource-constrained settings.

## Background

Childhood cancer is a major and growing public health challenge, particularly in low- and middle-income countries (LMICs), where the burden of disease and mortality remains disproportionately high. Although cancer in children is relatively rare compared with adult malignancies, an estimated 400,000 children and adolescents develop cancer each year worldwide, with over 90% of cases occurring in LMICs, where survival outcomes remain poor compared with high-income settings [1]. The 5-year survival rate for childhood cancer in high-income countries (HICs) frequently surpasses 80%, yet in many LMICs it remains below 30%, largely due to delayed diagnosis, limited access to specialised care and fragmented health systems [2]. Such disparities are amplified by challenges across the continuum of care, including insufficient diagnostic capacity, weak referral networks and socioeconomic barriers that delay presentation and treatment initiation, undermining child survival and contributing towards preventable morbidity and mortality [3].

Delays in childhood cancer care are conceptualised as a continuum encompassing patient, diagnostic, treatment and wider health-system delays, each contributing to prolonged time to definitive therapy. In HICs, integrated health systems with standardised referral protocols and strong primary care often achieve median intervals from symptom onset to therapy of less than 14 days; facilitating early diagnosis and improved outcomes [4]. In contrast, evidence from LMICs indicates that cumulative delays frequently extend beyond 1 to 3 months, with multiple systemic, geographic and socioeconomic determinants driving prolonged intervals. Prolonged delays not only increase the likelihood of advanced-stage presentation and more intensive therapy requirements but also elevate the risk of treatment complications and poor survival, particularly for solid tumours that often present with nonspecific and insidious symptoms [5].

Pakistan exemplifies the structural inequities inherent in many LMIC settings, where paediatric oncology services are highly centralised and concentrated in a small number of tertiary centres serving a population exceeding 240 million. Of this population, approximately 83 million are children aged 0–14 years, with an estimated 8,000 to 10,000 new paediatric cancer cases expected annually across the country. Despite these numbers, reported 5-year survival rates in Pakistan remain significantly lower than in high-income settings, ranging from 30% to 45% depending on the malignancy and institution [6, 7].

Furthermore, treatment abandonment remains a critical challenge in the national landscape, with institutional studies reporting abandonment rates between 15% and 25%, often driven by the same socioeconomic barriers that cause initial delays [8]. Families residing in rural and peri-urban areas often confront long travel distances, poor transport infrastructure and limited access to specialised diagnostics and paediatric oncology expertise, resulting in repeated healthcare consultations and delayed referral to appropriate care [9]. Moreover, low health literacy, high out-of-pocket health expenditures and competing priorities for daily subsistence compound delays, especially among socioeconomically vulnerable households [10]. These barriers contribute to advanced disease at presentation and hinder progress toward equitable cancer care outcomes.

Despite the pressing need to understand delays in childhood cancer pathways in Pakistan, empirical evidence remains fragmented and insufficient to guide policy and health-system reform. Existing studies have largely focused on isolated components of delay, such as diagnostic lag or physician delay, without capturing the full care pathway from symptom onset through health-system progression to treatment initiation [11]. There is a paucity of comprehensive, pathway-based analyses that quantify cumulative delays and assess independent determinants across patient-level, socioeconomic and health-system domains. Furthermore, the relative contributions of first point of healthcare contact, referral trajectories and centralised diagnostic services are poorly characterised, limiting the development of targeted, scalable and equity-oriented interventions designed to improve timely access to care.

Addressing these evidence gaps is essential given growing global initiatives such as the World Health Organisation’s Global Initiative for Childhood Cancer, which aims to improve survival rates and reduce disparities by 2030 through system strengthening and early diagnosis strategies [5]. Evidence from systematic reviews in LMICs highlights that traditional medicine use, household income, transportation limitations, rural residence, parental education and travel distance are among the most consistent determinants of delayed childhood cancer care, underscoring the multifactorial nature of this challenge [6]. Comprehensive, context-specific data are needed to inform priority setting, resource allocation and the design of effective public health interventions within Pakistan’s health system.

Therefore, this study provides a comprehensive, pathway-based assessment of delays in childhood cancer care in Pakistan. By quantifying patient, diagnostic, treatment and health-system delays among children with newly diagnosed malignancies, and identifying socio-demographic, clinical and system-level predictors of prolonged total delay, this work aims to generate actionable evidence for policy and practice. The findings are intended to guide strategies to decentralise diagnostic capacity, strengthen referral pathways, enhance primary care responsiveness and reduce socioeconomic barriers, thereby improving access to timely curative care and advancing equity for children with cancer in resource-constrained settings.

## Methods

This analytical cross-sectional study assessed delays in the initiation of cancer treatment among children with newly diagnosed malignancies. The study was conducted at the Paediatric Oncology Department of Children’s Hospital, Pakistan Institute of Medical Sciences (PIMS), Islamabad, Pakistan, from March to December 2025. All patients included in the analysis were diagnosed within this 2025 calendar period. PIMS is one of the country’s largest public sector tertiary-care centres, providing comprehensive paediatric oncology services. The hospital receives referrals from diverse urban and rural health facilities across Pakistan, enabling a comprehensive assessment of real-world patient pathways and health-system delays.

Children aged 0–14 years with a new diagnosis of malignancy who initiated definitive cancer-directed therapy (chemotherapy, surgery, radiotherapy or multimodal treatment) were eligible. This criterion ensured complete ascertainment of diagnostic and treatment timelines. Children who did not commence treatment due to refusal, abandonment prior to initiation, transfer to another facility without treatment or pursuit of alternative therapies were excluded due to incomplete delay data. A census sampling strategy was employed, enrolling all consecutively diagnosed and treated patients during the study period to minimise selection bias and enhance generalisability.

A structured data collection instrument was developed iteratively with input from paediatric oncologists, epidemiologists and health-systems researchers, supported by a focused review of international literature on childhood cancer delay metrics, particularly from LMICs. The final proforma captured demographic, clinical, behavioural and health-system variables necessary to quantify delay intervals and identify determinants. Data were obtained through caregiver interviews, medical records, referral documentation and hospital administrative databases.

Demographic and clinical variables included age, sex, parental education and monthly household income, area of residence, geographic region and cancer type. Health-seeking pathway variables included the first point of healthcare contact, number and type of facilities visited prior to diagnosis, referral trajectory and diagnostic processes. Caregiver awareness regarding childhood cancer and its curability was assessed as a proxy for health literacy. Caregiver-reported timelines were cross-validated with medical records whenever possible.

Delay intervals were conceptualised using a patient–health system continuum framework, allowing differentiation between caregiver-related and system-related barriers. All delays were measured in days using standardised reference points, as outlined in Table 1.

A threshold of >30 days for prolonged total delay was selected a priori based on published paediatric oncology delay literature and supported by the observed median total delay in this cohort [12]. All health-system contacts were verified using documentary evidence, including prescriptions, referral slips, imaging reports, laboratory records and clinic receipts. A comprehensive patient-level timeline was constructed for each participant, recording symptom onset, first healthcare contact, intermediate visits, arrival at tertiary care, diagnostic confirmation, multidisciplinary tumour board discussion and treatment initiation. Multiple data sources were triangulated to minimise recall bias and misclassification.

The study was approved by the Institutional Review Board of PIMS (Approval No. F.1-1/2015/ERB/SZAMBU/1392; Date: 20/02/2025). Written informed consent was obtained from parents or guardians. All data were anonymised, stored on secure password-protected servers and accessed only by authorised research personnel.

Data were double-entered and cross-validated. Continuous variables were summarised using means (standard deviation (SD)) or medians (interquartile range (IQR)), while categorical variables were presented as frequencies and percentages. Delay intervals were analysed both as continuous variables and as dichotomised outcomes. Bivariate analyses were conducted using chi-square (*χ*²), *t*-tests or Mann–Whitney *U* tests as appropriate. Multivariable logistic regression models were constructed to identify independent predictors of prolonged total delay, adjusting for demographic, socioeconomic, clinical and health-system variables identified a priori. Statistical significance was defined as a two-sided *p*-value <0.05.

## Results

The study analysed 71 children with a median age of 5 years (IQR 3–8) and a male predominance of 67.6%. The population showed significant socioeconomic vulnerability, with 71.8% residing in rural areas and 77.5% belonging to households with a monthly income below 50,000 PKR. While the majority of patients originated from various Pakistani provinces, 18.3% of the cohorts were Afghan nationals, consisting of both registered refugees and cross-border patients traveling on medical visas. Caregiver health literacy was profoundly limited; 87.3% had never heard of childhood malignancy, and only 11.3% were aware that paediatric cancer is curable (Table 2).

The median total delay from symptom onset to treatment initiation was 36 days (IQR 49), with 57.7% of children experiencing a prolonged delay exceeding the 30-day threshold. Health-system delay was the most significant contributor to this timeline, affecting 61.9% of patients with a median interval of 29 days. In contrast, patient-level delays exceeded 14 days in 46.5% of cases, while diagnostic and treatment delays were comparatively shorter, with medians of 13 and 4 days, respectively (Table 3).

The first point of healthcare contact strongly influenced total delay. Children initially presenting to private clinics, primary healthcare (PHC) facilities or district-level hospitals experienced longer delays (median 48–56 days) compared with direct presentation to tertiary centres (median 16.5 days) (Table 4).

Delays were significantly correlated with advanced disease progression and reduced survival (*p* < 0.05). In the solid tumour subgroup, 66.7% of children presented with advanced Stages III or IV disease. For those in the ‘critical delay’ group (>90 days), 50.0% presented with advanced-stage cancer compared to only 11.1% of those in the 0–30 day group. Clinical outcomes mirrored these trends, as the 12-month event-free survival (EFS) dropped from 83.3% for patients treated within 30 days to 60.0% for those experiencing delays exceeding 90 days (Table 5).

Multivariable logistic regression identified that the strongest independent predictor of prolonged total delay was an initial healthcare contact outside of a tertiary centre (aOR 3.96; 95% CI 1.82–8.63). Other significant socioeconomic predictors included low household income (aOR 3.09) and rural residence (aOR 2.42). Furthermore, children with solid tumours (aOR 2.67) and those whose caregivers lacked prior awareness of childhood cancer (aOR 2.85) were significantly more likely to face prolonged intervals before initiating definitive therapy (Table 6).

## Discussion

This study provides a comprehensive, pathway-based assessment of delays in childhood cancer care in Pakistan, elucidating how patient-level, diagnostic, treatment-related and health-system factors converge to prolong the interval from symptom onset to treatment initiation. More than half of children experienced a total delay exceeding 30 days, with a median of 36 days. This duration is clinically meaningful, as delays beyond 4–6 weeks are associated with advanced-stage disease at diagnosis, intensified treatment regimens and poorer survival, particularly for solid tumours [13, 14]. Importantly, delays did not stem from a single bottleneck; instead, they accumulated across multiple stages of care, reflecting a fragmented and highly centralised health system.

Patient-level delays were substantial, with nearly half of children (46.5%) experiencing delays exceeding 14 days from symptom onset to first healthcare contact. Low caregiver awareness was a major contributor, as only 13% had ever heard of childhood cancer and 11% knew it was curable. These levels are markedly lower than those reported in HICs [15] and align with findings from Lahore, Pakistan, where 81.7% of caregivers demonstrated limited understanding of childhood cancer [16]. Common presenting symptoms, including fever, pallor, bone pain, swelling or weight loss, were often misattributed to minor infections, nutritional deficiencies or trauma, delaying formal care-seeking. When care was sought, general practitioners (GPs) and private clinics frequently managed symptoms empirically without suspicion of malignancy, resulting in multiple consultations before referral patterns observed previously in Karachi, Pakistan [17] and Egypt [18].

Financial constraints further compounded patient delays. Out-of-pocket costs for repeated consultations, diagnostic investigations, travel and lost wages imposed substantial economic burden, often leading families to defer care or pursue alternative therapies such as herbal, homeopathic or spiritual remedies. Approximately 30%–50% of families reported using non-biomedical interventions prior to oncology referral [19, 20]. These findings underscore that delayed care-seeking reflects structural poverty and low health literacy rather than caregiver negligence. Strengthening PHC capacity and training GPs to recognise early warning signs of childhood cancer has been shown to reduce diagnostic delays in other LMIC settings [21, 22] and represents a critical intervention for Pakistan.

Diagnostic delays exceeding 21 days were observed in 25.4% of children, with a median interval of 13 days. Definitive diagnosis often requires histopathology, immunohistochemistry, cytogenetic analysis, bone marrow examination and advanced imaging such as computed tomography, magnetic resonance imaging and nuclear medicine scans. The specialised diagnostic services are concentrated in a limited number of tertiary facilities, including PIMS-Islamabad, resulting in prolonged waiting times, repeated hospital visits and additional financial burdens, particularly for families from remote regions [23, 24]. Strengthening PHC diagnostic capacity, introducing referral-linked vouchers and leveraging telepathology could meaningfully reduce these delays. Evidence from Lahore, Pakistan and India indicates that decentralisation of selected diagnostic services can substantially shorten diagnostic intervals [25, 26].

Treatment delays were comparatively limited, with only 18.3% of children initiating therapy more than 21 days after diagnosis and a median delay of 4 days. Once a confirmed diagnosis is made, treatment initiation within tertiary centres is generally timely. Nevertheless, capacity constraints, including limited operating theaters, radiotherapy slots, high patient load, intermittent chemotherapy supply and restricted blood products contributed to delays, reflecting systemic limitations rather than clinical indecision [4]. With fewer than a dozen dedicated paediatric oncology units serving over 240 million people in Pakistan, these bottlenecks underscore the urgent need for expanded and decentralised oncology capacity.

Health-system delays were the dominant contributor to prolonged total delay, affecting 61.9% of children with a median of 29 days. Fragmented referral pathways, coordination across multiple departments and repeated transitions through private clinics, PHC facilities and district hospitals introduced cumulative delays. Comparable referral-related delays have been documented in China, Bangladesh, Nepal and Tanzania [27–30], highlighting that systemic design rather than caregiver behaviour primarily drives prolonged intervals.

The observed correlation between delay intervals and clinical outcomes provides a critical benchmark for paediatric oncology in Pakistan. In this cohort, children initiating treatment within the 30-day standard achieved a 12-month EFS of 83.3%, which dropped precipitously to 60.0% for those experiencing critical delays exceeding 90 days. This survival gap is closely mirrored by disease progression; half of the solid tumour cases (50.0%) in the critical delay group presented with advanced Stages III or IV disease compared to only 11.1% in the timely group. These findings contrast sharply with outcomes in high-income settings where standardised referral pathways maintain much narrower delay windows and higher EFS [19]. The significance of this study lies in its ability to quantify how systemic fragmentation, specifically, first contact outside tertiary care (aOR 3.96), serves as a primary driver of advanced-stage presentation. Implementing decentralised diagnostic hubs and standardised ‘red-flag’ referral algorithms at the primary care level is essential to shift the presentation of childhood cancer from advanced, often incurable stages to earlier, treatable phases, thereby improving the national survival trajectory.

Socioeconomic and geographic factors further compounded delays. Children from rural areas or low-income households (< 50,000 PKR/month) were significantly more likely to experience prolonged total delays, reflecting poor transport infrastructure, long travel distances and financial barriers for multiple healthcare visits. These upstream determinants intersected with referral-related delays, resulting in median total delays of 36 days, with over 14% of children experiencing delays exceeding 90 days, placing them at high risk of advanced-stage disease and poor outcomes. Children with solid tumours experienced longer delays than those with hematological malignancies, consistent with prior studies [13, 18], likely due to nonspecific and insidious symptom presentation.

Decentralisation of diagnostic services through regional hubs, mobile pathology units and tele-oncology platforms could reduce dependence on tertiary centres. Standardised childhood cancer red-flag algorithms and mandatory referral timelines at PHC and district levels are urgently needed. Social protection measures, including travel subsidies, patient navigation services and income support during treatment initiation, have demonstrated reductions in both delays and treatment abandonment. Integrating childhood cancer awareness into maternal and child health programs provides a scalable and cost-effective strategy to improve early recognition and timely care.

## Strengths and limitations of the study

While prior studies in Pakistan have explored isolated aspects of diagnostic lag, physician delay or abandonment, this study represents one of the first comprehensive, pathway-based assessments in Northern Pakistan to quantify the cumulative burden from symptom onset through health-system progression to definitive therapy initiation. Limitations include the cross-sectional design, which restricts causal inference, and potential recall bias despite triangulation. Additionally, the relatively small sample size (*N* = 71) from a single centre may limit the statistical power to detect smaller effect sizes and the generalisability of the findings to all regions of Pakistan.

## Conclusion

Prolonged delays in childhood cancer care in Pakistan are driven by the convergence of geographic inequity, socioeconomic vulnerability and health-system fragmentation, rather than caregiver negligence. These delays are not inevitable but stem from modifiable structural factors. Addressing them through integrated referral systems, decentralised diagnostic services, strengthened PHC, caregiver education and social protection measures is critical to improving survival and advancing equity in childhood cancer outcomes. Implementing these interventions can serve as a model for other resource-constrained settings and underscores the importance of embedding childhood cancer care within universal health coverage and broader health-system strengthening agendas.

## Conflicts of interest

The authors declare no competing interests.

## Funding

This study did not receive any specific grant from funding agencies in the public, commercial or not-for-profit sectors.

## Ethical approval and informed consent

The study was approved by the Institutional Review Board of Pakistan Institute of Medical Sciences (PIMS), Islamabad (Approval No. F.1-1/2015/ERB/SZAMBU/1392). Written informed consent was obtained from the parents or legal guardians of all participating children prior to enrollment.

## Author contributions

**Ruqayya Manzoor and Nuzhat Yasmeen**: Conceptualisation and design of the study; principal investigator; oversight of data collection; interpretation of findings; critical revision of the manuscript for intellectual content; final approval of the version to be published; accountable for all aspects of the work.

**Junaid Jamshed**: Data management; database development; data cleaning and verification; statistical support; contribution to methods and results sections; manuscript review.

**Hijab Shaheen**: Data collection; caregiver interviews; clinical data abstraction; contribution to manuscript drafting.

**Ana Farooq**: Data collection; literature review; contribution to background and discussion sections.

**Nazia Rafique**: Data collection; verification of referral and diagnostic timelines; manuscript editing.

All authors have reviewed and approved the final manuscript, and agree to be accountable for the work.

## Figures and Tables

**Table 1.** Operational definitions of treatment delay intervals and criteria for prolonged delay.

Delay type	Operational definition
Patient delay	Time (days) from caregiver-reported symptom onset to first contact with any formal healthcare provider (public or private)
Health-system delay	Time (days) from first healthcare contact to completion of initial clinical assessment and referral to an appropriate cancer treatment facility
Diagnostic delay	Time (days) from first healthcare contact to confirmed cancer diagnosis, defined by histopathological verification or unequivocal radiological diagnosis
Treatment delay	Time (days) from confirmed cancer diagnosis to initiation of definitive cancer therapy (chemotherapy, surgery or multimodal treatment)
Total delay	Time (days) from symptom onset to initiation of definitive cancer treatment, representing the cumulative burden of patient- and health-system-related delays

**Table 2.** Baseline socio-demographic, socioeconomic and clinical characteristics of children with newly diagnosed cancer (N = 71).

Characteristic		n (%)
**Age, years**	0–4	31 (43.7)
	5–9	28 (39.4)
	10–14	12 (16.9)
Mean (SD): 5.8 (3.1); Median (IQR): 5 (3–8)		
**Sex**	Male	48 (67.6)
	Female	23 (32.4)
**Residence**	Rural	51 (71.8)
	Urban	20 (28.2)
**Region**	Islamabad	7 (9.9)
	Punjab	35 (49.3)
	Khyber Pakhtunkhwa (KPK)	12 (16.9)
	Azad Jammu & Kashmir (AJK)	15 (21.1)
	Gilgit-Baltistan (GB)	1 (1.4)
	Afghanistan	1 (1.4)
**Cancer category**	Leukemias	38 (53.5)
	Lymphomas	10 (14.1)
	Solid tumours	18 (25.4)
	Central nervous system (CNS) tumours	3 (4.2)
	Others	2 (2.8)
**Disease stage at presentation (Solid tumours, *n* = 18)**	Early (Stages I–II)	6 (33.3)
	Advanced (Stages III–IV)	12 (66.7)
**Maternal education**	No formal education	32 (45.1)
	Any formal education	39 (54.9)
**Paternal education**	No formal education	16 (22.5)
	Any formal education	55 (77.5)
**Monthly household income (PKR)**	< 50,000	55 (77.5)
	50,000–100,000	14 (19.7)
	> 100,000	2 (2.8)
**Ever heard of childhood malignancy**	Yes	9 (12.7)
	No	62 (88.3)
**Knows childhood cancer is curable**	Yes	8 (11.3)
	No	63 (88.7)

Mean age 5.8 years (SD 3.1); median age 5 years (IQR 3–8), PKR = Pakistani Rupees

**Table 3.** Distribution of patient, health-system, diagnostic, treatment and total delays.

Delay category	n (%)	Median (IQR)	Prolonged delay, n (%)
**Patient delay**			
0–7 days	29 (40.8)	13 (43)	33 (46.5)
8–14 days	9 (12.7)		
15–30 days	14 (19.7)		
31–60 days	12 (16.9)		
> 60 days	7 (9.9)		
**Healthcare system delay**			
0–7 days	6 (8.5)	29 (39)	44 (61.9)
8–14 days	13 (18.3)		
15–30 days	21 (29.6)		
> 30 days	31 (43.7)		
**Diagnostic delay**			
0–7 days	25 (35.2)	13 (17)	18 (25.4)
8–21 days	28 (39.4)		
22–45 days	15 (21.1)		
> 45 days	3 (4.2)		
**Treatment delay**			
0–7 days	48 (67.6)	4 (9)	13 (18.3)
8–14 days	9 (12.7)		
15–21 days	9 (12.7)		
> 21 days	4 (5.6)		
**Total delay**			
0–30 days	30 (42.3)	36 (49)	41 (57.7)
31–90 days	31 (43.7)		
> 90 days	10 (14.1)		

Prolonged delay defined as >30 days for total delay

**Table 4.** Total delay to treatment initiation by first point of healthcare contact.

First presentation site	n (%)	Median total delay (IQR), days
Private clinic/GP	31 (43.7%)	49 (63.5)
**PHC/RHC**	3 (4.2%)	48 (20.0)
**THQ/DHQ hospital**	9 (12.7%)	56 (41.0)
**Tertiary care hospital**	28 (39.4%)	16.5 (21.0)

RHC = Rural Health Centre; THQ = Tehsil Headquarters Hospital; DHQ = District Headquarters Hospital

**Table 5.** Clinical outcomes and disease staging stratified by total delay intervals (N = 71).

Total delay category	Advanced disease stage (III–IV)	12-month EFS
**0–30** d**ays (Standard)**	2 (11.1%)	25 (83.3%)
**31–90** d**ays (Prolonged)**	7 (38.9%)	22 (70.9%)
**> 90** d**ays (Critical)**	9 (50.0%)	6 (60.0%)

**Table 6.** Multivariable logistic regression of factors associated with prolonged total delay (> 30 days).

Predictor	Adjusted OR (95% CI)	p-value
Age > 5 years	1.49 (0.73–3.04)	0.27
Male sex	1.08 (0.54–2.15)	0.83
Rural residence	2.42 (1.16–5.03)	0.02
Low household income (< PKR 50,000)	3.09 (1.42–6.72)	0.01
No maternal education	2.18 (1.01–4.71)	0.05
No paternal education	2.22 (1.03–4.79)	0.041
First contact outside tertiary care	3.96 (1.82–8.63)	< 0.001
Solid tumours (vs leukemia/lymphoma)	2.67 (1.29–5.53)	0.01
No awareness of childhood cancer	2.85 (1.29–6.31)	0.01

Models adjusted for age, sex, cancer type, residence, parental education, household income, caregiver awareness and first healthcare contact. OR = odds ratio; CI = confidence interval
